# Involvement of Programmed Cell Death in Neurotoxicity of Metallic Nanoparticles: Recent Advances and Future Perspectives

**DOI:** 10.1186/s11671-016-1704-2

**Published:** 2016-11-03

**Authors:** Bin Song, Ting Zhou, Jia Liu, LongQuan Shao

**Affiliations:** 1 Guizhou Provincial People’s Hospital, Guiyang, 550002 China; 2 Nanfang Hospital, Southern Medical University, Guangzhou, 510515 China

**Keywords:** Brain, Apoptosis, Autophagy, Necroptosis, Pyroptosis, Nanoparticles

## Abstract

The widespread application of metallic nanoparticles (NPs) or NP-based products has increased the risk of exposure to NPs in humans. The brain is an important organ that is more susceptible to exogenous stimuli. Moreover, any impairment to the brain is irreversible. Recently, several in vivo studies have found that metallic NPs can be absorbed into the animal body and then translocated into the brain, mainly through the blood–brain barrier and olfactory pathway after systemic administration. Furthermore, metallic NPs can cross the placental barrier to accumulate in the fetal brain, causing developmental neurotoxicity on exposure during pregnancy. Therefore, metallic NPs become a big threat to the brain. However, the mechanisms underlying the neurotoxicity of metallic NPs remain unclear. Programmed cell death (PCD), which is different from necrosis, is defined as active cell death and is regulated by certain genes. PCD can be mainly classified into apoptosis, autophagy, necroptosis, and pyroptosis. It is involved in brain development, neurodegenerative disorders, psychiatric disorders, and brain injury. Given the pivotal role of PCD in neurological functions, we reviewed relevant articles and tried to summarize the recent advances and future perspectives of PCD involvement in the neurotoxicity of metallic NPs, with the purpose of comprehensively understanding the neurotoxic mechanisms of NPs.

## Review

### Introduction

With the rapid development of nanotechnology, metallic (metal or metal oxide) nanoparticles (NPs), with a diameter ranging from 1 to 100 nm, are used in cosmetics [1], food addictives [2], building industry [3], paints [4], battery [5], and biomedical applications [6], owing to their extraordinary physicochemical properties. Metallic NP-based products facilitate our daily life; however, it has several disadvantages. The widespread application of NP-based products increases the risk of exposure to metallic NPs for humans, especially for those who work in industries involving the use of these materials. [7]. In addition, several in vivo studies have demonstrated that once animals are exposed to metallic NPs through intravenous injection [8], intranasal instillation [9], oral administration [10], inhalation [11], and intraperitoneal injection [12], NPs can be absorbed into the body and then re-distributed into secondary organs, such as the brain, liver, spleen, lungs, and kidneys. The brain is an important organ susceptible to harmful substances [13–15], and impairment to the brain is irreversible. Accumulated metallic NPs, an exogenous stimuli, induce apoptosis, up-regulate inflammatory responses, activate signaling pathways, disturb the neurotransmitters, and impair organelles (such as mitochondria), and these changes contribute to the neurotoxicity of NPs, consequently leading to brain dysfunction. However, the mechanisms underlying the neurotoxicity of metallic NPs remain unclear.

Programmed cell death (PCD), which is different from necrosis, is defined as active cell death, which is regulated by certain genes. The role of PCD is to balance the proportion of dead cells and healthy cells and maintain homeostasis. In general, PCD can be classified into apoptosis, autophagy, necroptosis, and pyroptosis [16, 17]. PCD can either be observed under physiological conditions or be induced by exogenous stimuli. Moreover, PCD is reported to be closely related to brain development [18, 19], neurodegenerative disorders [20, 21], brain injury [22, 23], and psychiatric disorders [24]. Based on the important role of PCD in neurological functions, in this review, we summarized the recent advances and put forward some suggestions regarding the involvement of PCD in the neurotoxicity of metallic NPs by analyzing relevant articles. We expect that investigating the correlation between PCD and metallic NPs can help us to understand the mechanisms underlying the neurotoxicity of NPs completely.

### Accumulation of Metallic NPs in the Brain After Systemic Administration

The brain is the main target of metallic NPs, and brain damage is irreversible. Therefore, more attention should be paid to the threat posed by metallic NPs on brain health. Metallic NPs can be absorbed into the body and then translocated into the brain, mainly through the blood–brain barrier (BBB) and direct nose-to-brain (or olfactory) pathway bypassing the BBB.

The results of several in vivo studies have revealed that metallic NPs can be detected in animal brain after systemic administration. Rats showed higher anxious index, which indicated impaired neurobehavioral functions, and the contents of TiO_2_ NPs in the brain, lungs, and liver were elevated, owing to TiO_2_ NP exposure via intraperitoneal injection every 2 days for 20 days [12]. The concentrations of TiO_2_ NPs in the brain, liver, spleen, kidneys, lungs, and heart were elevated after the rats were exposed to NPs through single or repeated intravenous injection [8]. When the rats were exposed to silver NPs through chronic intranasal instillation, the brain subunits including the cortex, hippocampus, cerebellum, olfactory bulb, and medulla exhibited higher contents of NPs [9]. The exposure to silver NPs through single intravenous injection can also increase the NP levels in the mouse brain [25, 26]. Meanwhile, an oral administration was able to increase the content of silver NPs in the rat brains as well [10, 27–29].

In addition to TiO_2_ and silver NPs, exposure to gold NPs through single intravenous injection increased the concentration of NPs in the mouse brain [30]. Inhalation administration for 15 days led to elevated gold NP levels in the subunits of the brain including the olfactory bulb, hippocampus, striatum, frontal cortex, entorhinal cortex, septum, and cerebellum [11]. The gold NP content in the rat brain was enhanced 24 h after the intravenous injection [31]. Zinc oxide (ZnO) NP exposure through repeated oral administration slightly increased the ZnO NP levels in the rat brains [32]. The content of copper (Cu) NPs in the olfactory bulb increased after the mice were chronically exposed to Cu NPs through intranasal instillation [33, 34].

Even worse, the metallic NPs can cross the placental barrier and accumulate in fetal organs on exposure to NPs during pregnancy. When pregnant mice were intravenously injected with silica and TiO_2_ NPs, both NPs were detected in the fetal brain, fetal liver, and placenta 24 h after the injection. These changes might impair the fetal development including neurodevelopment, which indicates that developmental neurotoxicity can be induced by silica and TiO_2_ NPs [35]. We must pay much attention to the neurodevelopmental toxicity of metallic NPs, as fetal brain is more susceptible to harmful stimuli.

To sum up, investigating the bio-distribution of metallic NPs might help us to screen the safest metallic nanomaterials and administration routes that can protect the brain from being affected by NPs. Therefore, relevant studies should be further performed in the future. In addition, studies should be conducted to comprehensively investigate the relationship between exposure to metallic NPs during pregnancy and fetal brain development.

### The Contribution of PCD to the Neurotoxicity of Metallic NPs

As mentioned above, metallic NPs can be translocated into the brain after systemic administration. This accumulation in turn can lead to neurotoxicity. PCD as an active cell death process mainly consists of apoptosis, autophagy, necroptosis, and pyroptosis. Moreover, PCD plays an important role in neurological functions. Therefore, we will discuss the correlation between PCD and the neurotoxicity of metallic NPs, with the purpose of comprehensively understanding the neurotoxic mechanisms of NPs.

#### Apoptosis—Established Role in the Neurotoxicity of Metallic NPs

**Fig. 1:**
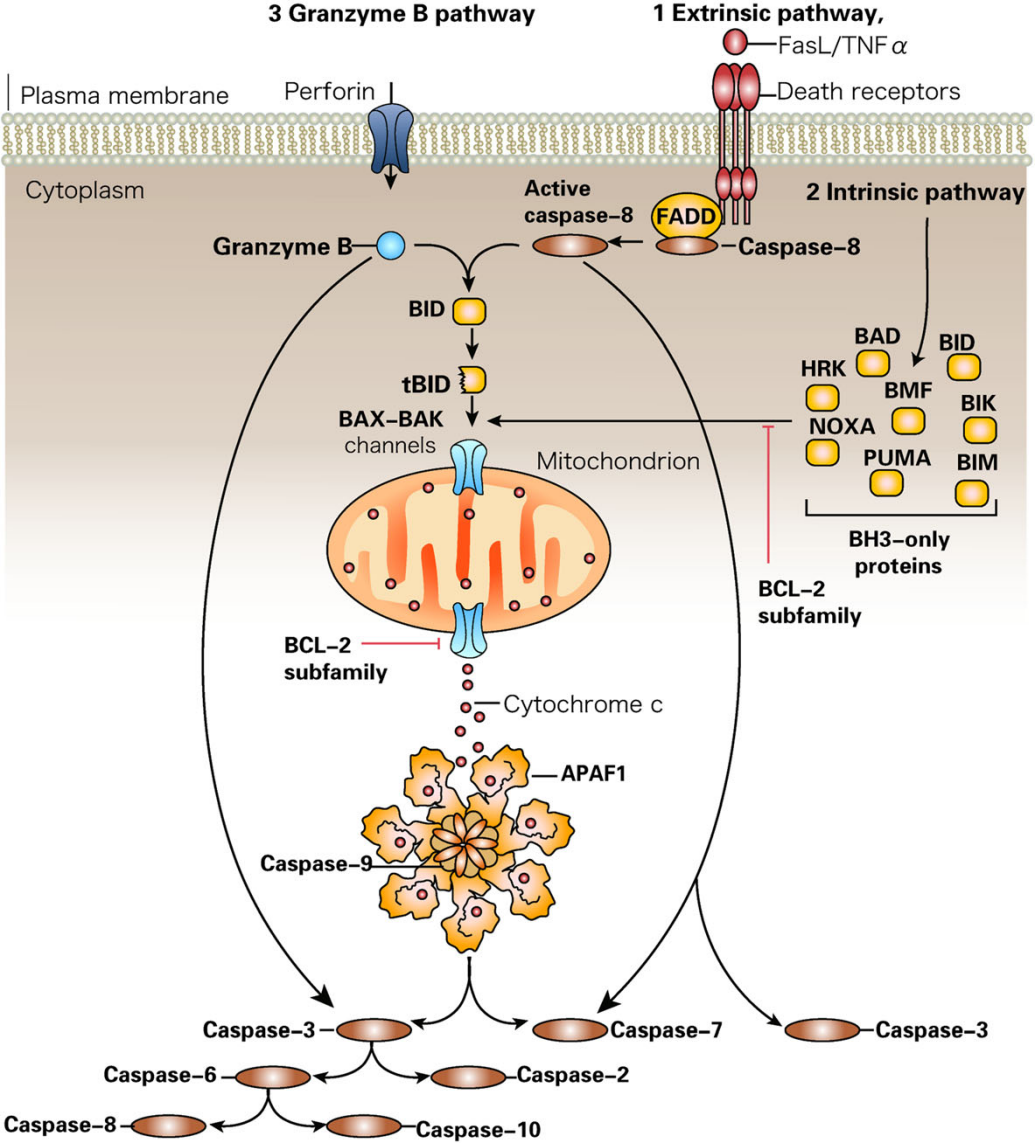
Caspase activation pathways [131]. Caspase activation by the extrinsic pathway (route 1) involves the binding of extracellular death ligands (such as FasL or tumor necrosis factor-α (TNFα)) to transmembrane death receptors. Engagement of death receptors with their cognate ligands provokes the recruitment of adaptor proteins, such as the Fas-associated death domain protein (FADD), which in turn recruit and aggregate several molecules of caspase-8, thereby promoting its autoprocessing and activation. Active caspase-8 then proteolytically processes and activates caspase-3 and caspase-7, provoking further caspase activation events that culminate in substrate proteolysis and cell death. In some situations, extrinsic death signals can crosstalk with the intrinsic pathway through caspase-8-mediated proteolysis of the BH3-only protein BID (BH3-interacting domain death agonist). Truncated BID (tBID) can promote mitochondrial cytochrome *c* release and assembly of the apoptosome (comprising ~7 molecules of apoptotic protease-activating factor-1 (APAF1) and the same number of caspase-9 homodimers). In the intrinsic pathway (route 2), diverse stimuli that provoke cell stress or damage typically activate one or more members of the BH3-only protein family. BH3-only proteins act as pathway-specific sensors for various stimuli and are regulated in distinct ways. BH3-only protein activation above a crucial threshold overcomes the inhibitory effect of the anti-apoptotic B cell lymphoma-2 (BCL-2) family members and promotes the assembly of BAK–BAX oligomers within mitochondrial outer membranes. These oligomers permit the efflux of intermembrane space proteins, such as cytochrome *c*, into the cytosol. On release from mitochondria, cytochrome *c* can seed apoptosome assembly. Active caspase-9 then propagates a proteolytic cascade of further caspase activation events. The granzyme B-dependent route to caspase activation (route 3) involves the delivery of this protease into the target cell through specialized granules that are released from cytotoxic T lymphocytes (CTL) or natural killer (NK) cells. CTL and NK granules contain numerous granzymes as well as a poreforming protein, perforin, which oligomerizes in the membranes of target cells to permit entry of the granzymes. Granzyme B, similar to the caspases, also cleaves its substrates after Asp residues and can process BID as well as caspase-3 and caspase-7 to initiate apoptosis. *BAD* BCL-2 antagonist of cell death, *BAK* BCL-2-antagonist/killer-1, *BAX* BCL-2-associated X protein, *BID* BH3-interacting domain death agonist, *BIK* BCL-2-interacting killer, *BIM* BCL-2-like-11, *BMF* BCL-2 modifying factor, *HRK* harakiri (also known as death protein-5), *PUMA* BCL-2 binding component-3

Apoptosis is the first and most commonly studied PCD type. It can be simply defined as programmed “self-killing” [36]. Apoptosis plays an important role in cell renovation and elimination of injured cells. Dysregulation of cell apoptosis can induce cell death and impairment of tissues, consequently leading to organ dysfunction [37]. Human health and diseases can be regulated by cell apoptosis [37, 38]. Apoptosis is mediated by caspase-dependent pathways (Fig. 1) [39, 40]. Generally, apoptosis is characterized by blebbing, DNA fragmentation, and caspase activation [41–43].

##### In Vitro Studies Related to Apoptosis in Neurotoxicity of Metallic NPs

Long et al. first reported that TiO_2_ NPs can induce apoptosis in immortalized mouse microglia (BV2), rat dopaminergic neuronal cells (N27), and primary embryonic rat stratum neurons [44]. Another research group revealed that the proportion of apoptotic cells in human astrocyte-like cell lines (U87) increased on TiO_2_ NP exposure [45]. TiO_2_ NP exposure inhibited cell proliferation, increased the proportion of apoptotic cells, activated caspase-3 in rat (C6) and human (U373) glial cell lines, and was accompanied by hyper-condensed nuclei [46]. TiO_2_ NPs can also attenuate cell viability and increase the number of apoptotic cells after the exposure of murine microglia cell lines (N9) to NPs [47]. Besides, the mitochondrial membrane potential (MMP) of PC12 cells was reduced by TiO_2_ NP exposure, whereas the proportion of apoptotic cells was enhanced. The expression of Bax and p53 was up-regulated, while that of Bcl-2 was down-regulated at the protein level. Meanwhile, TiO_2_ NP exposure promoted the activity of caspase-3 [48]. These changes indicated that apoptosis in PC12 cells was induced by TiO_2_ NPs. Cell viability was not reduced by TiO_2_ NPs; however, the cell cycle was disturbed on exposure to TiO_2_ NPs. An increased proportion of apoptotic cells and up-regulated MMP were observed in human neuronal cell lines (SH-SY5Y) [49].

After the rat primary cortical neurons were exposed to silver NP, cell viability decreased, the protein levels of caspase-3 increased, the proportion of apoptotic cells increased, and a DNA ladder was observed. These findings suggested that silver NP induced apoptosis, which led to neurotoxicity [50]. Silver NP can reduce cell viability, increase the number of apoptotic cells, enhance reactive oxygen species (ROS) production, and activate caspase-3 in rat primary neurons. In vivo experiments further confirmed that silver NP can be detected in the rat brain, and after intranasal administration, they can in turn up-regulate the protein levels of caspase-3, which was consistent with the results observed in the in vitro experiment. These findings suggested that silver NP exposure can induce caspase-dependent apoptosis contributing to neurotoxicity [51]. Another study revealed that silver NPs reduced cell viability and increased the number of apoptotic cells in PC12, which further verified the role of caspases in apoptosis. Co-treating PC12 cells with caspase-8 or caspase-9 inhibitors attenuated the apoptosis induced by NPs, which suggested that both death receptor-regulated signaling and mitochondrial-mediated pathway are involved in silver NP-induced apoptosis in PC12 cells [52]. The proportion of apoptotic cells in rat primary astrocytes, determined by using DNA fragmentation assay [53] and flow cytometry [54], was elevated in the silver NP-treated group. The caspase activity was also enhanced in this group [54].

When the human neuroblastoma cell lines (SH-SY5Y) were treated with ZnO NPs, cell viability was reduced, swelling or loss of organelles was detected, the number of apoptotic cells increased, and the activities of caspase-3/7 were up-regulated [55]. A similar conclusion was reached by another study, which revealed that the number of apoptotic cells in SH-SY5Y was enhanced by ZnO NPs [56]. Mouse neural stem cells (NSCs) can be impaired by ZnO NPs. Cell viability was attenuated, accompanied by impaired morphology (such as membrane blebbing and hyper-condensed chromatin) and increased proportion of apoptotic cells, which indicated that apoptosis was induced by ZnO NPs [57]. After C6 cells were exposed to ZnO NPs, the NPs were taken up by the C6 cells, which resulted in the reduction of cell viability. Meanwhile, apoptotic-like morphological features such as blebbing, nucleus shrinkage, and hyper-condensed chromatin were observed. The number of apoptotic cells increased as well [58]. ZnO NPs induced apoptosis in U87 cells, which was characterized by condensed chromatin, nuclear fragmentation, and the increase in the apoptotic cell proportion [59].

The neurotoxicity induced by TiO_2_, silver, and ZnO NPs was widely investigated. In addition, other metallic nanomaterials were able to induce apoptosis in neuronal cells.

Viability of PC12 was reduced, and the number of apoptotic cells was elevated by copper NPs [60]. Iron oxide NPs can also decrease the cell viability and induce apoptosis in PC12 cells, characterized by the increased proportion of apoptotic cells and up-regulated protein levels of p53 and Bax, as well as down-regulated Bcl-2 protein expression [61].

##### In Vivo Studies Related to Apoptosis in the Neurotoxicity of Metallic NPs

TiO_2_ NPs can be detected in the mouse hippocampus after intragastric treatment for 60 days. The accumulated TiO_2_ NPs can in turn induce apoptotic-like changes in cell morphology (such as condensed chromatin and shrinkage of the nuclear membrane) and DNA ladder. Meanwhile, the expression of caspase-3, caspase-9, Bax, and cytochrome c was up-regulated accompanied by the down-regulated expression of Bcl-2 at gene and protein levels. These findings suggested that an intrinsic apoptosis pathway in the mouse hippocampus was induced by TiO_2_ NP exposure, which resulted in impaired spatial recognition ability [62]. TiO_2_ NPs can be detected in the rat brain after intravenous injection, once a week for 4 weeks. This accumulation increased the number of apoptotic cells, induced DNA ladder, activated caspase-3, up-regulated the expressions of p53, Bax, and cytochrome c, and down-regulated Bcl-2 expression at gene and protein levels. These results indicated that mitochondria-mediated apoptosis in the rat brain was induced by TiO_2_ NPs [63]. After the mice were exposed to TiO_2_ NPs for 90 consecutive days through intranasal instillation, NPs were detected in the mouse brain. Meanwhile, the proportion of apoptotic cells in the hippocampus increased, as indicated by apoptotic morphology (shrinkage of the nucleus, condensed chromatin, and swollen mitochondria), and the expression of genes related to apoptosis, determined by DNA microarray analysis, was altered [64]. Exposure to TiO_2_ NPs during pregnancy via subcutaneous injection can alter the expression of genes related to apoptosis, which were determined by DNA microarray analysis in the brain of mouse offsprings [65]. These changes indicated that TiO_2_ NP exposure could induce developmental neurotoxicity.

Silver NPs can increase the number of apoptotic cells in the rat hippocampal subunits (CA1, CA2, CA3, and DG) after oral administration for 28 days [66]. Exposure to silver NPs during pregnancy can also elevate the proportion of apoptotic cells in the hippocampal subunits of rat offsprings [67]. Meanwhile, silver NPs can be taken up by human embryonic neural precursor cells (HNPCs), thereby inducing apoptosis in HNPCs [68]. These findings indicated that apoptosis was also probably implicated in the developmental neurotoxicity of silver NPs.

Rats exposed to CuO NPs through intraperitoneal injection once a day for 14 days performed poorly in the Morris water maze (MWM) test and long-term potentiation (LTP) was affected, which indicated that the rat hippocampus was impaired by NPs. At the same time, both the activity of caspase-3 and the levels of 4-hydroxynonenal (HNE) in the rat hippocampus were up-regulated in the NP-treated group when compared to that in the control group, indicating an apoptotic process in the hippocampal zone [69]. Apoptosis induced by gold NPs also resulted in neurotoxicity. A study involving in vivo and in vitro experiments demonstrated that gold NP exposure activated caspase-3 and increased the number of apoptotic SH-SY5Y cells. The in vivo experiment showed that gold NPs can be detected in the mouse brain and can promote caspase-3 activity after intravenous injection, which was consistent with the results of the in vitro experiment [70]. Aluminum oxide NP exposure through intranasal instillation damaged the mouse neurobehavioral function, accompanied by reduced MMP, increased apoptotic cells, and up-regulated caspase-3 gene expression [71] in the mouse brain.

##### Regulation of Neuronal Apoptosis Induced by Metallic NPs

Although apoptosis was involved in the neurotoxicity of metallic NPs, the molecular mechanisms by which the NPs regulated apoptosis are unknown. Studies showed that oxidative stress (OS) status was related to cell apoptosis [72–74]. Therefore, a few rescue studies were conducted to verify the role of NP-induced OS in nanoneurotoxicity. TiO_2_ NP exposure decreased cell viability and increased ROS production in PC12 cells; it also increased the proportion of apoptotic PC12 cells. However, pretreating PC12 cells with N-acetylcysteine (NAC) can reverse these changes. These findings suggested that the apoptosis in PC12 was probably mediated by TiO_2_ NP-induced ROS [75], as NAC had an antioxidant property [76, 77]. Treatment with ZnO NPs can lead to decreased cell viability, excessive ROS production, and apoptotic morphology, such as nuclear shrinkage in primary astrocytes. Meanwhile, the reduction in MMP suggested that the intrinsic apoptotic pathway was implicated in neurotoxicity. Further experiments found that the proportion of apoptotic astrocytes increased. At the same time, the expression of Bax, cleaved poly-ADP-ribose polymerase (PARP), and cleaved caspase-3 was up-regulated at the protein level. However, the level of Bcl-2 protein decreased on treatment with ZnO NPs. Pretreatment of astrocytes with NAC or Jun N-terminal kinase (JNK) inhibitor could reverse the harmful effects induced by metallic NPs, which indicated that apoptosis was probably caused by NP-induced ROS through the JNK pathway [78]. ZnO NPs can reduce PC12 viability and increase the number of apoptotic PC12 cells, which was determined using flow cytometry. However, pretreating PC12 cells with N-(mercaptopropionyl)-glycine (N-MPG) can lead to the inhibition of apoptotic process, which indicated that apoptosis in PC12 might be mediated by ZnO NP-induced OS [79]. N-MPG is another type of ROS scavenger [80]. Copper oxide (CuO) NPs can decrease the mouse hippocampal cell line (HT22) viability and increase the number of apoptotic HT22; they also up-regulated Bax gene levels and down-regulated Bcl-2 mRNA levels in HT22. Meanwhile, the OS status in HT22 cells was disrupted. However, pretreating HT22 cells with crocetin can attenuate those harmful impacts. These findings indicated that CuO NP-induced apoptosis in HT22 cells was probably mediated by NP-induced OS [81]. Crocetin possessed antioxidant and neuroprotective capabilities and could counteract OS [82–84]. These results indicated that apoptosis was most probably initiated by metallic NP-induced OS. However, more rescue studies are needed to further confirm it. In addition to OS mechanism, other potential mechanisms should be investigated.

The findings from the above-mentioned in vitro and in vivo studies demonstrated that metallic NP-induced apoptosis was involved in the neurotoxicity of NPs. Meanwhile, a few rescue studies revealed that apoptosis in neurotoxicity was probably regulated by metallic NP-induced OS. In addition, findings were mostly obtained from in vitro studies. Furthermore, except TiO_2_ NPs, ZnO NPs, and silver NPs, other metallic nanomaterials were less studied. Besides, metallic NPs can cross the placental barrier to affect fetal brain development, but studies about the involvement of apoptosis in developmental neurotoxicity of metallic NPs were scarce.

#### Autophagy—Role in the Neurotoxicity of Metallic NPs Needs Further Verification

**Fig. 2:**
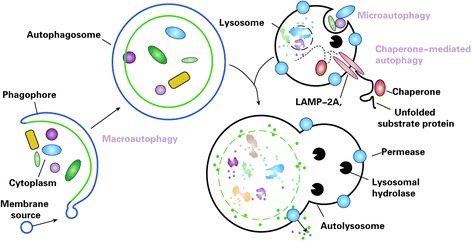
Different types of autophagy [132]. Microautophagy refers to the sequestration of cytosolic components directly by lysosomes through invaginations in their limiting membrane. The function of this process in higher eukaryotes is not known, whereas microautophagy-like processes in fungi are involved in selective organelle degradation. In the case of macroautophagy, the cargoes are sequestered within a unique double-membrane cytosolic vesicle, an autophagosome. Sequestration can be either nonspecific, involving the engulfment of bulk cytoplasm, or selective, targeting specific cargoes such as organelles or invasive microbes. The autophagosome is formed by expansion of the phagophore, but the origin of the membrane is unknown. Fusion of the autophagosome with an endosome (not shown) or a lysosome provides hydrolases. Lysis of the autophagosome inner membrane and breakdown of the contents occurs in the autolysosome, and the resulting macromolecules are released back into the cytosol through membrane permeases. Chaperone-mediated autophagy (CMA) involves direct translocation of unfolded substrate proteins across the lysosome membrane through the action of a cytosolic and lysosomal chaperone hsc70, and the integral membrane receptor lysosome-associated membrane protein type 2A (LAMP-2A)

Recently, autophagy has become a hot topic and has attracted much attention. It can be simply defined as programmed “self-eating” [36]. Autophagy is different from apoptosis, and is mediated by caspase-independent pathways. It can be identified as a particular accommodation of cells to starvation. The process of autophagy includes cell degradation, in which the cargo in the cytoplasm is transported into the lysosome. Autophagy is a dynamic recycling system and it can maintain cellular renovation and homeostasis [85]. It can be classified into microautophagy, macroautophagy, and chaperone-mediated autophagy (CMA) (Fig. 2) [86, 87].

Many studies revealed that metallic NPs can induce autophagy in non-neuronal cells including human keratinocytes (HaCaT) [88], normal lung cells [89], MRC-5 fibroblasts [90], immune cells [91], human hepatocellular carcinoma HepG2 cells [92], murine peritoneal macrophage cells (RAW264.7) [93], and mouse embryonic fibroblasts [94]. Therefore, autophagy has been regarded as one of the mechanisms underlying nanotoxicity [95]. Moreover, it has been reported that autophagy was involved in neurotoxicity [96–98]. A few studies on the role of autophagy in the neurotoxicity of metallic NPs have been published. Kenzaoui et al. [99] found that the exposure of human cerebral endothelial cells (HCECs) to aminoPVA (poly(vinyl alcohol/vinylamine))-coated ultrasmall superparamagnetic iron oxide (USPIO) NPs, autophagic vacuoles were observed in HCECs and LC3-II. In addition, the cathepsin D protein levels were up-regulated, which suggested that autophagy in HCECs was induced by NPs. Manshian et al. [100] treated murine C17.2 neural progenitor cells with silver NPs and found that the LC3 fluorescent intensity was enhanced, which indicated that autophagy in C17.2 was induced by silver NPs. Since OS can induce autophagy [101–103], which was implicated in the neurotoxicity of metallic NPs [104–106], the role of autophagy in the neurotoxicity of metallic NPs should be not ignored.

#### Necroptosis and Pyroptosis—Potential Role in the Neurotoxicity of Metallic NPs

**Fig. 3:**
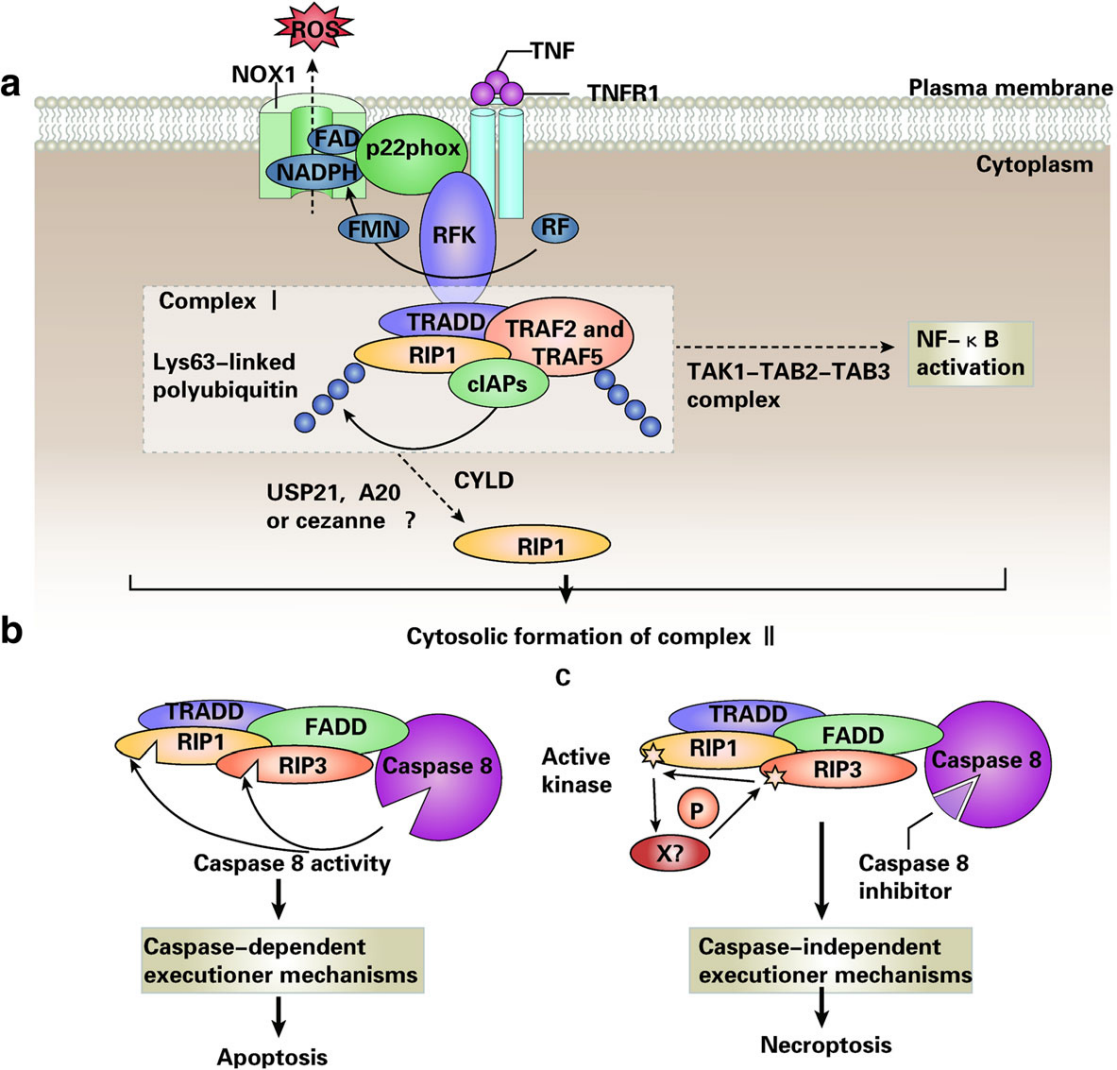
TNFR1-elicited signaling pathways [133]. **a** On tumor necrosis factor (TNF) binding, TNF receptor 1 (TNFR1) undergoes a conformational change, allowing for the intracellular assembly of the so-called TNFR complex I, which includes TNF receptor-associated death domain (TRADD), receptor-interacting protein 1 (RIP1; also known as RIPK1), cellular inhibitor of apoptosis proteins (cIAPs), TNF receptor-associated factor 2 (TRAF2) and TRAF5. On cIAP-mediated Lys63-ubiquitylation, RIP1 can serve as a scaffold for the recruitment of transforming growth factor-β activated kinase 1 (TAK1) and TAK1-binding protein 2 (TAB2) and TAB3, which initiate the canonical nuclear factor-κB (NF-κB) activation pathway. Riboflavin kinase (RFK) physically bridges the TNFR1 death domain to p22phox (also known as CYBA), the common subunit of multiple NADPH oxidases, including NADPH oxidase 1 (NOX1), which also contributes to TNFα-induced necroptosis by generating reactive oxygen species (ROS). Conversely, on deubiquitylation by cylindromatosis (CYLD; and perhaps also by A20 (also known as TNFAIP3), cezanne (also known as OTUD7B) or ubiquitin-specific peptidase 21 (USP21)), RIP1 exerts lethal functions, which can be executed by two distinct types of cell death. **b** The internalization of TNFR1 is accompanied by a change in its binding partners that leads to the cytosolic assembly of TNFR complex II, which often (but not invariably) contains TRADD, FAS-associated protein with a death domain (FADD), caspase-8, RIP1, and RIP3 (also known as RIPK3). Normally, caspase-8 triggers apoptosis by activating the classical caspase cascade. It also cleaves, and hence inactivates, RIP1 and RIP3. **c** If caspase-8 is blocked by pharmacological or genetic interventions, RIP1 and RIP3 become phosphorylated (perhaps by an unidentified kinase) and engage the effector mechanisms of necroptosis. *FAD* flavin adenine nucleotide, *FMN* flavin mononucleotide

The role of necroptosis and pyroptosis in the toxicity of metallic NPs has not been extensively studied. Necroptosis, which can also be called “programmed necrosis,” is initiated by activating the death receptor with stimuli (Fig. 3). Receptor-interacting protein kinases 1 and 3 are frequently involved in necroptosis [107, 108]. Studies on the relationship between necroptosis and nanotoxicity are rare. However, recent studies have demonstrated that cigarette can induce necroptosis in the mouse airway [109], carbon tetrachloride can lead to liver fibrosis via necroptosis [110], and glutamate can induce necroptosis in HT-22 cells [111].

**Fig. 4:**
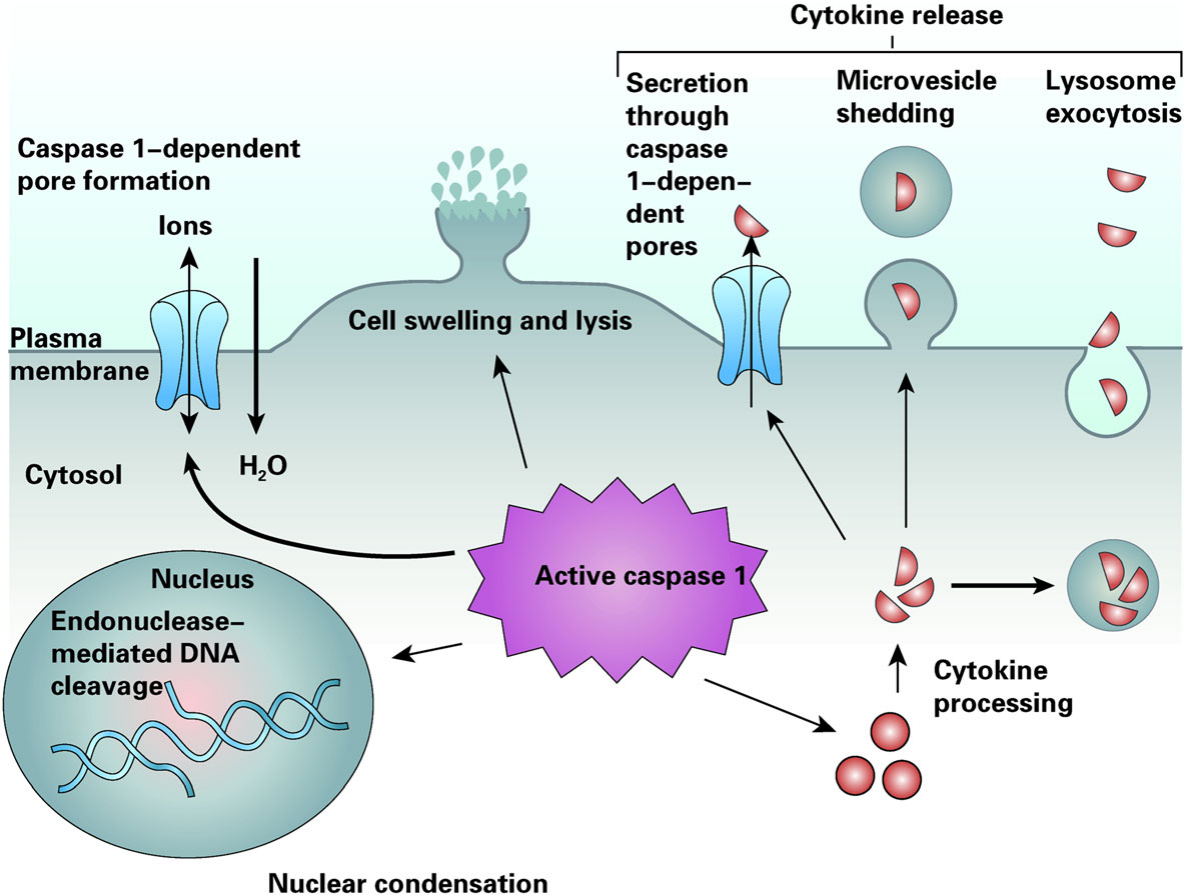
Pyroptosis, an inflammatory host response [134]. Caspase-1 is cleaved and activated in response to multiple stimuli, but once activated, caspase-1 results in a conserved program of cell death referred to as pyroptosis. Caspase-1 activation also leads to rapid formation of plasma-membrane pores with a diameter of 1.1–2.4 nm. These pores dissipate cellular ionic gradients, allowing water influx, cell swelling, and osmotic lysis. The pro-forms of interleukin-1β (IL-1β) and IL-18 are processed by caspase-1 and released during pyroptosis, although the exact mechanism of secretion remains controversial. Secretion does not require lysis and is temporally associated with caspase-1-dependent pore formation, suggesting that these pores facilitate cytokine release. Other suggested secretion mechanisms include caspase-1-independent lysosome exocytosis and microvesicle shedding. Caspase-1 activity results in cleavage of chromosomal DNA by an unidentified endonuclease. Cleavage of DNA does not result in the oligonucleosomal fragments observed during apoptosis. Nuclear condensation is also observed but nuclear integrity is maintained, unlike the nuclear fragmentation observed during apoptosis

Pyroptosis, a new type of PCD, is typically regulated by the caspase-1-dependent signaling pathway (Fig. 4). Caspase-1 is not involved in apoptosis or autophagy [112, 113]. It has been reported that silver NPs can induce pyroptosome formation in human monocytes (THP-1) and up-regulate the caspase-1 protein expression, which indicates that pyroptosis in THP-1 was induced by NPs [114].

The role of necroptosis and pyroptosis in the neurotoxicity of metallic NPs is uncertain. However, several reports have demonstrated that necroptosis and pyroptosis can be induced by OS [115–118], and metallic NP-induced OS contributed to neurotoxicity. Furthermore, necroptosis was involved in neurotoxicity induced by other harmful substances, such as iron [119], *Streptococcus pneumoniae* [120], and TNF-α [121]. Therefore, we hypothesized that metallic NP-induced OS can probably initiate necroptosis and pyroptosis, which might contribute to the neurotoxicity of NPs.

### Future Perspectives

Based on the results of the above-mentioned studies, we put forward some suggestions for future research to understand the role of PCD in the neurotoxicity of metallic NPs completely.

1. Since the role of apoptosis in the neurotoxicity of metallic NPs has been widely studied, the signaling pathways through which NPs regulate neuronal apoptosis should be investigated comprehensively.
2. As metallic NP exposure during pregnancy can affect fetal brain development [122–124], much attention should be paid to the role of apoptosis in developmental neurotoxicity induced by NPs.
3. More in vivo studies are needed to further confirm the vital role of apoptosis in the neurotoxicity of metallic NPs.
4. In addition to TiO_2_ NPs and silver NPs that were most widely studied, other metallic nanomaterials including NPs of gold, copper, copper oxide, aluminum oxide, and iron oxide should be investigated to understand the pivotal role of apoptosis in the neurotoxicity of NPs completely.
5. Several studies have already been performed to investigate the role of autophagy in non-neuronal cells [125–127], and autophagy was implicated in brain/neuron damage [128–130]. Although a few studies confirmed the involvement of autophagy in the neurotoxicity of metallic NPs, its role in neurotoxicity still needs further verification.
6. Whether necroptosis or pyroptosis is involved in the neurotoxicity of metallic NPs should be investigated in the future.
7. The correlation among apoptosis, autophagy, necroptosis, and pyroptosis in the neurotoxicity of metallic NPs should be studied.

## Conclusions

The widespread application of metallic NP-based products raises concerns about the safety of NPs. The brain is the most important organ that can be impaired by metallic NPs. Based on the vital role of PCD in neurological functions, we summarized articles related to the role of PCD in the neurotoxicity of metallic NPs, and we found that apoptosis was involved in the neurotoxicity of metallic NPs. Although autophagy is involved in nanotoxicity, few studies on the relationship between autophagy and neurotoxicity of metallic NPs have been reported. In addition, studies about the role of necroptosis or pyroptosis in the neurotoxicity of metallic NPs are scarce. Therefore, for unraveling the neurotoxic mechanisms underlying metallic NPs, the role of PCD in nanoneurotoxicity should be investigated comprehensively in the future.

## Abbreviations

APAF1: Apoptotic protease-activating factor-1
BBB: Blood–brain barrier
cIAPs: Cellular inhibitor of apoptosis proteins
CMA: Chaperone-mediated autophagy
CTL: Cytotoxic T lymphocytes
Cu: Copper
CuO: Copper oxide
ER: Endoplasmic reticulum
FADD: Fas-associated death domain protein
HCECs: Human cerebral endothelial cells
HNE: Hydroxynoneal
HNPCs: Human embryonic neural precursor cells
IL-1β: Interleukin-1β
JNK: Jun N-terminal kinase
LAMP-2A: Lysosome-associated membrane protein type 2A
LTP: Long-term potentiation
MMP: Mitochondrial membrane potential
MWM: Morris water maze
NAC: N-acetylcysteine
NF-κB: Nuclear factor-κB
NK: Natural killer
N-MPG: N-(mercaptopropionyl)-glycine
NOX1: NADPH oxidase 1
NPs: Nanoparticles
NSCs: Neural stem cells
OS: Oxidative stress
PARP: Poly-ADP-ribose polymerase
PCD: Programmed cell death
RFK: Riboflavin kinase
RIP1: Receptor-interacting protein 1
ROS: Reactive oxygen species
TAB2: TAK1-binding protein 2
TAK1: Transforming growth factor-β activated kinase 1
TNFα: Tumor necrosis factor-α
TRADD: TNF receptor-associated death domain
TRAF2: TNF receptor-associated factor 2
USP21: Ubiquitin-specific peptidase 21
USPIO: Ultrasmall superparamagnetic iron oxide
ZnO: Zinc oxide

## References

[1] Wu X, Guy RH (2009). Applications of nanoparticles in topical drug delivery and in cosmetics. J. Drug Delivery Sci. Technol..

[2] Shi LE, Li ZH, Zheng W, Zhao YF, Jin YF, Tang ZX (2014). Synthesis, antibacterial activity, antibacterial mechanism and food applications of ZnO nanoparticles: a review. Food Addit Contam Part A Chem Anal Control Expo Risk Assess.

[3] Sang L, Zhao Y, Burda C (2014). TiO2 nanoparticles as functional building blocks. Chem Rev.

[4] Kumar A, Vemula PK, Ajayan PM, John G (2008). Silver-nanoparticle-embedded antimicrobial paints based on vegetable oil. Nat Mater.

[5] Ma L, Hendrickson KE, Wei SY, Archer LA (2015). Nanomaterials: science and applications in the lithium-sulfur battery. Nano Today.

[6] Sasidharan A, Monteiro-Riviere NA (2015). Biomedical applications of gold nanomaterials: opportunities and challenges. Wiley Interdiscip. Rev. Nanomed. Nanobiotechnol..

[7] Pelclova D, Zdimal V, Fenclova Z, Vlckova S, Turci F, Corazzari I (2016). Markers of oxidative damage of nucleic acids and proteins among workers exposed to TiO2 (nano) particles. Occup Environ Med.

[8] Geraets L, Oomen AG, Krystek P, Jacobsen NR, Wallin H, Laurentie M et al (2014) Tissue distribution and elimination after oral and intravenous administration of different titanium dioxide nanoparticles in rats. Part Fibre Toxicol 11. doi:10.1186/1743-8977-11-30 10.1186/1743-8977-11-30 PMC4105399 24993397

[9] Wen RX, Yang XX, Hu LG, Sun C, Zhou QF, Jiang GB (2016). Brain-targeted distribution and high retention of silver by chronic intranasal instillation of silver nanoparticles and ions in Sprague-Dawley rats. J Appl Toxicol.

[10] Espinosa-Cristobal LF, Martinez-Castanon GA, Loyola-Rodriguez JP, Patino-Marin N, Reyes-Macias JF, Vargas-Morales JM et al (2013) Toxicity, distribution, and accumulation of silver nanoparticles in Wistar rats. J Nanopart Res 15(6). doi:10.1007/s11051-013-1702-6

[11] Balasubramanian SK, Poh KW, Ong CN, Kreyling WG, Ong WY, Yu LE (2013). The effect of primary particle size on biodistribution of inhaled gold nano-agglomerates. Biomaterials.

[12] Ben Younes NR, Amara S, Mrad I, Ben-Slama I, Jeljeli M, Omri K (2015). Subacute toxicity of titanium dioxide (TiO2) nanoparticles in male rats: emotional behavior and pathophysiological examination. Environ Sci Pollut Res.

[13] Landrigan PJ, Sonawane B, Butler RN, Trasande L, Callan R, Droller D (2005). Early environmental origins of neurodegenerative disease in later life. Environ Health Perspect.

[14] Bowler RM, Gysens S, Diamond E, Nakagawa S, Drezgic M, Roels HA (2006). Manganese exposure: neuropsychological and neurological symptoms and effects in welders. Neurotoxicology.

[15] Rohlman DS, Lasarev M, Anger WK, Scherer J, Stupfel J, McCauley L (2007). Neurobehavioral performance of adult and adolescent agricultural workers. Neurotoxicology.

[16] Nagata S (2005) DNA degradation in development and programmed cell death. Annu Rev Immunol 23:853–75. doi:10.1146/annurev.immunol.23.021704.115811 10.1146/annurev.immunol.23.021704.115811 15771588

[17] Ouyang L, Shi Z, Zhao S, Wang FT, Zhou TT, Liu B (2012). Programmed cell death pathways in cancer: a review of apoptosis, autophagy and programmed necrosis. Cell Prolif.

[18] Buss RR, Sun W, Oppenheim RW (2006) Adaptive roles of programmed cell death during nervous system development. Annu Rev Neurosci 29:1–35. doi:10.1146/annurev.neuro.29.051605.112800 10.1146/annurev.neuro.29.051605.112800 16776578

[19] Yamaguchi Y, Miura M (2015). Programmed cell death in neurodevelopment. Dev Cell.

[20] Ghavami S, Shojaeid S, Yeganeh B, Ande SR, Jangamreddy JR, Mehrpour M (2014). Autophagy and apoptosis dysfunction in neurodegenerative disorders. Prog Neurobiol.

[21] Krantic S, Mechawar N, Reix S, Quirion R (2005). Molecular basis of programmed cell death involved in neurodegeneration. Trends Neurosci.

[22] Zhang XP, Chen YM, Jenkins LW, Kochanek PM, Clark RSB (2005). Bench-to-bedside review: apoptosis/programmed cell death triggered by traumatic brain injury. Crit Care.

[23] Li LZ, Bao YJ, Zhao M (2011). 17Beta-estradiol attenuates programmed cell death in cortical pericontusional zone following traumatic brain injury via upregulation of ERalpha and inhibition of caspase-3 activation. Neurochem Int.

[24] Niculescu AB, Levey DF, Phalen PL, Le-Niculescu H, Dainton HD, Jain N (2015). Understanding and predicting suicidality using a combined genomic and clinical risk assessment approach. Mol Psychiatry.

[25] Recordati C, De Maglie M, Bianchessi S, Argentiere S, Cella C, Mattiello S et al (2016) Tissue distribution and acute toxicity of silver after single intravenous administration in mice: nano-specific and size-dependent effects. Part Fibre Toxicol 13: doi:10.1186/s12989-016-0124-x 10.1186/s12989-016-0124-x PMC4772516 26926244

[26] Dziendzikowska K, Gromadzka-Ostrowska J, Lankoff A, Oczkowski M, Krawczynska A, Chwastowska J (2012). Time-dependent biodistribution and excretion of silver nanoparticles in male Wistar rats. J Appl Toxicol.

[27] van der Zande M, Vandebriel RJ, Van Doren E, Kramer E, Rivera ZH, Serrano-Rojero CS (2012). Distribution, elimination, and toxicity of silver nanoparticles and silver ions in rats after 28-day oral exposure. ACS Nano.

[28] Loeschner K, Hadrup N, Qvortrup K, Larsen A, Gao XY, Vogel U et al (2011) Distribution of silver in rats following 28 days of repeated oral exposure to silver nanoparticles or silver acetate. Part Fibre Toxicol 8: doi:10.1186/1743-8977-8-18 10.1186/1743-8977-8-18 PMC3123173 21631937

[29] Park EJ, Bae E, Yi J, Kim Y, Choi K, Lee SH (2010). Repeated-dose toxicity and inflammatory responses in mice by oral administration of silver nanoparticles. Environ Toxicol Pharmacol.

[30] Lee JK, Kim TS, Bae JY, Jung AY, Lee SM, Seok JH (2015). Organ-specific distribution of gold nanoparticles by their surface functionalization. J Appl Toxicol.

[31] De Jong WH, Hagens WI, Krystek P, Burger MC, Sips A, Geertsma RE (2008). Particle size-dependent organ distribution of gold nanoparticles after intravenous administration. Biomaterials.

[32] Cho WS, Kang BC, Lee JK, Jeong J, Che JH, Seok SH (2013) Comparative absorption, distribution, and excretion of titanium dioxide and zinc oxide nanoparticles after repeated oral administration. Part Fibre Toxicol 10: doi:10.1186/1743-8977-10-9 10.1186/1743-8977-10-9 PMC3616827 23531334

[33] Zhang LL, Bai R, Liu Y, Meng L, Li B, Wang LM (2012). The dose-dependent toxicological effects and potential perturbation on the neurotransmitter secretion in brain following intranasal instillation of copper nanoparticles. Nanotoxicology.

[34] Liu Y, Gao YX, Zhang LL, Wang TC, Wang JX, Jiao F (2009). Potential health impact on mice after nasal instillation of nano-sized copper particles and their translocation in mice. J Nanosci Nanotechnol.

[35] Yamashita K, Yoshioka Y, Higashisaka K, Mimura K, Morishita Y, Nozaki M (2011). Silica and titanium dioxide nanoparticles cause pregnancy complications in mice. Nat Nanotechnol.

[36] Maiuri MC, Zalckvar E, Kimchi A, Kroemer G (2007). Self-eating and self-killing: crosstalk between autophagy and apoptosis. Nat Rev Mol Cell Biol.

[37] Elmore S (2007). Apoptosis: a review of programmed cell death. Toxicol Pathol.

[38] Wirawan E, Vande Walle L, Kersse K, Cornelis S, Claerhout S, Vanoverberghe I et al (2010) Caspase-mediated cleavage of Beclin-1 inactivates Beclin-1-induced autophagy and enhances apoptosis by promoting the release of proapoptotic factors from mitochondria. Cell Death Dis 1: doi:10.1038/cddis.2009.16 10.1038/cddis.2009.16 PMC3032505 21364619

[39] Fan TJ, Han LH, Cong RS, Liang J (2005). Caspase family proteases and apoptosis. Acta Biochim Biophys Sin.

[40] Riedl SJ, Shi YG (2004). Molecular mechanisms of caspase regulation during apoptosis. Nat Rev Mol Cell Biol.

[41] Kanter M, Unsal C, Aktas C, Erboga M (2016). Neuroprotective effect of quercetin against oxidative damage and neuronal apoptosis caused by cadmium in hippocampus. Toxicol Ind Health.

[42] Liu ZG, Song G, Zou C, Liu GG, Wu WQ, Yuan T (2015). Acrylamide induces mitochondrial dysfunction and apoptosis in BV-2 microglial cells. Free Radic Biol Med.

[43] Ogaly HA, Khalaf AA, Ibrahim MA, Galal MK, Abd-Elsalam RM (2015). Influence of green tea extract on oxidative damage and apoptosis induced by deltamethrin in rat brain. Neurotoxicol Teratol.

[44] Long TC, Tajuba J, Sama P, Saleh N, Swartz C, Parker J (2007). Nanosize titanium dioxide stimulates reactive oxygen species in brain microglia and damages neurons in vitro. Environ Health Perspect.

[45] Lai JCK, Lai MB, Jandhyam S, Dukhande VV, Bhushan A, Daniels CK (2008). Exposure to titanium dioxide and other metallic oxide nanoparticles induces cytotoxicity on human neural cells and fibroblasts. Int J Nanomed.

[46] Marquez-Ramirez SG, Delgado-Buenrostro NL, Chirino YI, Iglesias GG, Lopez-Marure R (2012). Titanium dioxide nanoparticles inhibit proliferation and induce morphological changes and apoptosis in glial cells. Toxicology.

[47] Li XB, Xu SQ, Zhang ZR, Schluesener HJ (2009). Apoptosis induced by titanium dioxide nanoparticles in cultured murine microglia N9 cells. Chin Sci Bull.

[48] Wu J, Sun JA, Xue Y (2010). Involvement of JNK and P53 activation in G2/M cell cycle arrest and apoptosis induced by titanium dioxide nanoparticles in neuron cells. Toxicol Lett.

[49] Valdiglesias V, Costa C, Sharma V, Kilic G, Pasaro E, Teixeira JP (2013). Comparative study on effects of two different types of titanium dioxide nanoparticles on human neuronal cells. Food Chem Toxicol.

[50] Kim SH, Ko JW, Koh SK, Lee IC, Son JM, Moon C (2014). Silver nanoparticles induce apoptotic cell death in cultured cerebral cortical neurons. Mol Cell Toxicol.

[51] Yin NY, Liu Q, Liu JY, He B, Cui L, Li ZN (2013). Silver nanoparticle exposure attenuates the viability of rat cerebellum granule cells through apoptosis coupled to oxidative stress. Small.

[52] Hadrup N, Loeschner K, Mortensen A, Sharma AK, Qvortrup K, Larsen EH (2012). The similar neurotoxic effects of nanoparticulate and ionic silver in vivo and in vitro. Neurotoxicology.

[53] Salazar-Garcia S, Silva-Ramirez AS, Ramirez-Lee MA, Rosas-Hernandez H, Rangel-Lopez E, Castillo CG et al (2015) Comparative effects on rat primary astrocytes and C6 rat glioma cells cultures after 24-h exposure to silver nanoparticles (AgNPs). J Nanopart Res 17(11). doi:10.1007/s11051-015-3257-1

[54] Sun C, Yin NY, Wen RX, Liu W, Jia YX, Hu LG (2016). Silver nanoparticles induced neurotoxicity through oxidative stress in rat cerebral astrocytes is distinct from the effects of silver ions. Neurotoxicology.

[55] Kim JH, Jeong MS, Kim DY, Her S, Wie MB (2015). Zinc oxide nanoparticles induce lipoxygenase-mediated apoptosis and necrosis in human neuroblastoma SH-SY5Y cells. Neurochem Int.

[56] Valdiglesias V, Costa C, Kilic G, Costa S, Pasaro E, Laffon B (2013). Neuronal cytotoxicity and genotoxicity induced by zinc oxide nanoparticles. Environ Int.

[57] Deng XY, Luan QX, Chen WT, Wang YL, Wu MH, Zhang HJ et al (2009) Nanosized zinc oxide particles induce neural stem cell apoptosis. Nanotechnology 20(11). doi:10.1088/0957-4484/20/11/115101 10.1088/0957-4484/20/11/115101 19420431

[58] Sruthi S, Mohanan PV (2015). Investigation on cellular interactions of astrocytes with zinc oxide nanoparticles using rat C6 cell lines. Colloid Surf B-Biointerfaces.

[59] Wahab R, Kaushik NK, Verma AK, Mishra A, Hwang IH, Yang YB (2011). Fabrication and growth mechanism of ZnO nanostructures and their cytotoxic effect on human brain tumor U87, cervical cancer HeLa, and normal HEK cells. J Biol Inorg Chem.

[60] Xu PJ, Xu J, Liu SC, Ren GG, Yang Z (2012) In vitro toxicity of nanosized copper particles in PC12 cells induced by oxidative stress. J Nanopart Res 14(6). doi:10.1007/s11051-012-0906-5

[61] Wu J, Ding TT, Sun J (2013). Neurotoxic potential of iron oxide nanoparticles in the rat brain striatum and hippocampus. Neurotoxicology.

[62] Hu R, Zheng L, Zhang T, Gao G, Cui Y, Cheng Z (2011). Molecular mechanism of hippocampal apoptosis of mice following exposure to titanium dioxide nanoparticles. J Hazard Mater.

[63] Meena R, Kumar S, Paulraj R (2015) Titanium oxide (TiO2) nanoparticles in induction of apoptosis and inflammatory response in brain. J Nanopart Res 17(1). doi:10.1007/s11051-015-2868-x

[64] Ze Y, Hu R, Wang X, Sang X, Ze X, Li B (2014). Neurotoxicity and gene-expressed profile in brain-injured mice caused by exposure to titanium dioxide nanoparticles. J Biomed Mater Res Part A.

[65] Shimizu M, Tainaka H, Oba T, Mizuo K, Umezawa M, Takeda K (2009) Maternal exposure to nanoparticulate titanium dioxide during the prenatal period alters gene expression related to brain development in the mouse. Part Fibre Toxicol 6. doi:10.1186/1743-8977-6-20 10.1186/1743-8977-6-20 PMC2726979 19640265

[66] Bagheri-abassi F, Alavi H, Mohammadipour A, Motejaded F, Ebrahimzadeh-bideskan A (2015). The effect of silver nanoparticles on apoptosis and dark neuron production in rat hippocampus. Iran J Basic Med Sci.

[67] Ataei ML, Ebrahimzadeh-bideskan AR (2014). The effects of nano-silver and garlic administration during pregnancy on neuron apoptosis in rat offspring hippocampus. Iran J Basic Med Sci.

[68] Soderstjerna E, Johansson F, Klefbohm B, Johansson UE (2013) Gold- and silver nanoparticles affect the growth characteristics of human embryonic neural precursor cells. PLoS One 8(3). doi:10.1371/journal.pone.0058211 10.1371/journal.pone.0058211 PMC3594300 23505470

[69] An L, Liu SC, Yang Z, Zhang T (2012). Cognitive impairment in rats induced by nano-CuO and its possible mechanisms. Toxicol Lett.

[70] Imperatore R, Carotenuto G, Di Grazia MA, Ferrandino I, Palomba L, Mariotti R (2015). Imidazole-stabilized gold nanoparticles induce neuronal apoptosis: an in vitro and in vivo study. J Biomed Mater Res Part A.

[71] Zhang QL, Li MQ, Ji JW, Gao FP, Bai R, Chen CY (2011). In vivo toxicity of nano-alumina on mice neurobehavioral profiles and the potential mechanisms. Int J Immunopathol Pharmacol.

[72] Hildeman DA (2004). Regulation of T-cell apoptosis by reactive oxygen species. Free Radic Biol Med.

[73] Zhao ZQ (2004). Oxidative stress-elicited myocardial apoptosis during reperfusion. Curr Opin Pharmacol.

[74] Mates JM, Segura JA, Alonso FJ, Marquez J (2008). Intracellular redox status and oxidative stress: implications for cell proliferation, apoptosis, and carcinogenesis. Arch Toxicol.

[75] Liu SC, Xu LJ, Zhang T, Ren GG, Yang Z (2010). Oxidative stress and apoptosis induced by nanosized titanium dioxide in PC12 cells. Toxicology.

[76] Dekhuijzen PNR (2004). Antioxidant properties of N-acetylcysteine: their relevance in relation to chronic obstructive pulmonary disease. Eur Respir J.

[77] Kamboj SS, Vasishta RK, Sandhir R (2010). N-acetylcysteine inhibits hyperglycemia-induced oxidative stress and apoptosis markers in diabetic neuropathy. J Neurochem.

[78] Wang JT, Deng XB, Zhang F, Chen DL, Ding WJ (2014) ZnO nanoparticle-induced oxidative stress triggers apoptosis by activating JNK signaling pathway in cultured primary astrocytes. Nanoscale Res Lett 9. doi:10.1186/1556-276x-9-117 10.1186/1556-276X-9-117 PMC3995614 24624962

[79] Zhao JX, Yao Y, Liu SC, Zhang T, Ren GG, Yang Z (2012) Involvement of reactive oxygen species and high-voltage-activated calcium currents in nanoparticle zinc oxide-induced cytotoxicity in vitro. J Nanopart Res 14(11). doi:10.1007/s11051-012-1238-1

[80] Yum S, Park H, Hong S, Jeong S, Kim W, Jung Y (2014). N-(2-mercaptopropionyl)-glycine, a diffusible antioxidant, activates HIF-1 by inhibiting HIF prolyl hydroxylase-2: Implication in amelioration of rat colitis by the antioxidant. Biochem Biophys Res Commun.

[81] Niska K, Santos-Martinez MJ, Radomski MW, Inkielewicz-Stepniak I (2015). CuO nanoparticles induce apoptosis by impairing the antioxidant defense and detoxification systems in the mouse hippocampal HT22 cell line: protective effect of crocetin. Toxicol Vitro.

[82] Yoshino F, Yoshida A, Umigai N, Kubo K, Lee MCI (2011). Crocetin reduces the oxidative stress induced reactive oxygen species in the stroke-prone spontaneously hypertensive rats (SHRSPs) brain. J Clin Biochem Nutr.

[83] Shen XC, Qian ZY (2006). Effects of crocetin on antioxidant enzymatic activities in cardiac hypertrophy induced by norepinephrine in rats. Pharmazie.

[84] Ahmad AS, Ansari MA, Ahmad M, Saleem S, Yousuf S, Hoda MN (2005). Neuroprotection by crocetin in a hemi-parkinsonian rat model. Pharmacol Biochem Behav.

[85] Mizushima N, Komatsu M (2011). Autophagy: renovation of cells and tissues. Cell.

[86] Klionsky DJ (2005). The molecular machinery of autophagy: unanswered questions. J Cell Sci.

[87] Massey AC, Zhang C, Cuervo AM (2006). Chaperone-mediated autophagy in aging and disease. Curr Top Dev Biol.

[88] Lopes VR, Loitto V, Audinot JN, Bayat N, Gutleb AC, Cristobal S (2016) Dose-dependent autophagic effect of titanium dioxide nanoparticles in human HaCaT cells at non-cytotoxic levels. J Nanobiotechnol 14. doi:10.1186/s12951-016-0174-0 10.1186/s12951-016-0174-0 PMC4802894 27001369

[89] Yu KN, Chang SH, Park SJ, Lim J, Lee J, Yoon TJ et al (2015) Titanium dioxide nanoparticles induce endoplasmic reticulum stress-mediated autophagic cell death via mitochondria-associated endoplasmic reticulum membrane disruption in normal lung cells. PLoS One 10(6). doi:10.1371/journal.pone.0131208 10.1371/journal.pone.0131208 PMC4485469 26121477

[90] Voicu SNP, Dinu D, Sima C, Hermenean A, Ardelean A, Codrici E (2015). Silica nanoparticles induce oxidative stress and autophagy but not apoptosis in the MRC-5 cell line. Int J Mol Sci.

[91] Johnson BM, Fraietta JA, Gracias DT, Hope JL, Stairiker CJ, Patel PR (2015). Acute exposure to ZnO nanoparticles induces autophagic immune cell death. Nanotoxicology.

[92] Yu YB, Duan JC, Yu Y, Li Y, Liu XM, Zhou XQ (2014). Silica nanoparticles induce autophagy and autophagic cell death in HepG2 cells triggered by reactive oxygen species. J Hazard Mater.

[93] Park EJ, Choi DH, Kim Y, Lee EW, Song J, Cho MH (2014). Magnetic iron oxide nanoparticles induce autophagy preceding apoptosis through mitochondrial damage and ER stress in RAW264.7 cells. Toxicol Vitro.

[94] Lee YH, Cheng FY, Chiu HW, Tsai JC, Fang CY, Chen CW (2014). Cytotoxicity, oxidative stress, apoptosis and the autophagic effects of silver nanoparticles in mouse embryonic fibroblasts. Biomaterials.

[95] Cohignac V, Landry MJ, Boczkowski J, Lanone S (2014). Autophagy as a possible underlying mechanism of nanomaterial toxicity. Nanomaterials.

[96] Luo J (2014). Autophagy and ethanol neurotoxicity. Autophagy.

[97] Gonzalez-Polo RA, Niso-Santano M, Ortiz-Ortiz MA, Gomez-Martin A, Moran JM, Garcia-Rubio L (2007). Inhibition of paraquat-induced autophagy accelerates the apoptotic cell death in neuroblastoma SH-SY5Y cells. Toxicol Sci.

[98] Nopparat C, Porter JE, Ebadi M, Govitrapong P (2010). The mechanism for the neuroprotective effect of melatonin against methamphetamine-induced autophagy. J Pineal Res.

[99] Kenzaoui BH, Bernasconi CC, Guney-Ayra S, Juillerat-Jeanneret L (2012). Induction of oxidative stress, lysosome activation and autophagy by nanoparticles in human brain-derived endothelial cells. Biochem J.

[100] Manshian BB, Pfeiffer C, Pelaz B, Heimerl T, Gallego M, Moller M (2015). High-content imaging and gene expression approaches to unravel the effect of surface functionality on cellular interactions of silver nanoparticles. ACS Nano.

[101] Chen Y, McMillan-Ward E, Kong J, Israels SJ, Gibson SB (2008). Oxidative stress induces autophagic cell death independent of apoptosis in transformed and cancer cells. Cell Death Differ.

[102] Scherz-Shouval R, Shvets E, Fass E, Shorer H, Gil L, Elazar Z (2007). Reactive oxygen species are essential for autophagy and specifically regulate the activity of Atg4. Embo J.

[103] Chen Y, Azad MB, Gibson SB (2009). Superoxide is the major reactive oxygen species regulating autophagy. Cell Death Differ.

[104] Rahman MF, Wang J, Patterson TA, Saini UT, Robinson BL, Newport GD (2009). Expression of genes related to oxidative stress in the mouse brain after exposure to silver-25 nanoparticles. Toxicol Lett.

[105] Long TC, Saleh N, Tilton RD, Lowry GV, Veronesi B (2006). Titanium dioxide (P25) produces reactive oxygen species in immortalized brain microglia (BV2): implications for nanoparticle neurotoxicity. Environ Sci Technol.

[106] Huerta-Garcia E, Perez-Arizti JA, Marquez-Ramirez SG, Delgado-Buenrostro NL, Chirino YI, Iglesias GG (2014). Titanium dioxide nanoparticles induce strong oxidative stress and mitochondrial damage in glial cells. Free Radic Biol Med.

[107] Wu W, Liu P, Li JY (2012). Necroptosis: an emerging form of programmed cell death. Crit. Rev. Oncol. Hematol..

[108] Linkermann A, Green DR (2014). Necroptosis. N. Engl. J. Med..

[109] Pouwels SD, Zijlstra GJ, van der Toorn M, Hesse L, Gras R, ten Hacken NHT (2016). Cigarette smoke-induced necroptosis and DAMP release trigger neutrophilic airway inflammation in mice. Am. J. Physiol. Lung Cell. Mol. Physiol..

[110] Choi HS, Kang JW, Lee SM (2015). Melatonin attenuates carbon tetrachloride-induced liver fibrosis via inhibition of necroptosis. Transl Res.

[111] Xu XS, Chua CC, Zhang M, Geng DQ, Liu CF, Hamdy RC (2010). The role of PARP activation in glutamate-induced necroptosis in HT-22 cells. Brain Res.

[112] Fink SL, Cookson BT (2005). Apoptosis, pyroptosis, and necrosis: mechanistic description of dead and dying eukaryotic cells. Infect Immun.

[113] Fink SL, Cookson BT (2006). Caspase-1-dependent pore formation during pyroptosis leads to osmotic lysis of infected host macrophages. Cell Microbiol.

[114] Simard JC, Vallieres F, de Liz R, Lavastre V, Girard D (2015). Silver nanoparticles induce degradation of the endoplasmic reticulum stress sensor activating transcription factor-6 leading to activation of the NLRP-3 inflammasome. J. Biol. Chem..

[115] Shindo R, Kakehashi H, Okumura K, Kumagai Y, Nakano H (2013). Critical contribution of oxidative stress to TNF alpha-induced necroptosis downstream of RIPK1 activation. Biochem Biophys Res Commun.

[116] Pacheco FJ, Almaguel FG, Evans W, Rios-Colon L, Filippov V, Leoh LS (2014). Docosahexanoic acid antagonizes TNF-alpha-induced necroptosis by attenuating oxidative stress, ceramide production, lysosomal dysfunction, and autophagic features. Inflamm Res.

[117] Song KJ, Jang YS, Lee YA, Kim KA, Lee SK, Shin MH (2011). Reactive oxygen species-dependent necroptosis in Jurkat T cells induced by pathogenic free-living Naegleria fowleri. Parasite Immunol.

[118] Jang Y, Lee AY, Jeong SH, Park KH, Paik MK, Cho NJ (2015). Chlorpyrifos induces NLRP3 inflammasome and pyroptosis/apoptosis via mitochondrial oxidative stress in human keratinocyte HaCaT cells. Toxicology.

[119] Dai MC, Zhong ZH, Sun YH, Sun QF, Wang YT, Yang GY (2013). Curcumin protects against iron induced neurotoxicity in primary cortical neurons by attenuating necroptosis. Neurosci Lett.

[120] Kim JY, Paton JC, Briles DE, Rhee DK, Pyo S (2015). Streptococcus pneumoniae induces pyroptosis through the regulation of autophagy in murine microglia. Oncotarget.

[121] Liu S, Wang X, Li Y, Xu L, Yu XL, Ge L (2014). Necroptosis mediates TNF-induced toxicity of hippocampal neurons. Biomed Res Int.

[122] Umezawa M, Tainaka H, Kawashima N, Shimizu M, Takeda K (2012). Effect of fetal exposure to titanium dioxide nanoparticle on brain development—brain region information. J Toxicol Sci.

[123] Cui YH, Chen XY, Zhou Z, Lei Y, Ma MN, Cao RJ (2014). Prenatal exposure to nanoparticulate titanium dioxide enhances depressive-like behaviors in adult rats. Chemosphere.

[124] Takahashi Y, Mizuo K, Shinkai Y, Oshio S, Takeda K (2010). Prenatal exposure to titanium dioxide nanoparticles increases dopamine levels in the prefrontal cotex and neostriatum of mice. J Toxicol Sci.

[125] Ebato C, Uchida T, Arakawa M, Komatsu M, Ueno T, Komiya K (2008). Autophagy is important in islet homeostasis and compensatory increase of beta cell mass in response to high-fat diet. Cell Metab.

[126] Ding WX, Li M, Chen XY, Ni HM, Lin CW, Gao WT (2010). Autophagy reduces acute ethanol-induced hepatotoxicity and steatosis in mice. Gastroenterology.

[127] Chen ZH, Kim HP, Sciurba FC, Lee SJ, Feghali-Bostwick C, Stolz DB et al (2008) Egr-1 Regulates autophagy in cigarette smoke-induced chronic obstructive pulmonary disease. PLoS One 3(10). doi:10.1371/journal.pone.0003316 10.1371/journal.pone.0003316 PMC2552992 18830406

[128] Shi RY, Weng JQ, Zhao L, Li XM, Gao TM, Kong JM (2012). Excessive autophagy contributes to neuron death in cerebral ischemia. CNS Neurosci Ther.

[129] Tizon B, Sahoo S, Yu H, Gauthier S, Kumar AR, Mohan P et al (2010) Induction of autophagy by cystatin C: a mechanism that protects murine primary cortical neurons and neuronal cell lines. PLoS One 5(3). doi:10.1371/journal.pone.0009819 10.1371/journal.pone.0009819 PMC2843718 20352108

[130] Lee SJ, Cho KS, Koh JY (2009). Oxidative injury triggers autophagy in astrocytes: the role of endogenous zinc. Glia.

[131] Taylor RC, Cullen SP, Martin SJ (2008). Apoptosis: controlled demolition at the cellular level. Nat Rev Mol Cell Biol.

[132] Mizushima N, Levine B, Cuervo AM, Klionsky DJ (2008). Autophagy fights disease through cellular self-digestion. Nature.

[133] Vandenabeele P, Galluzzi L, Vanden Berghe T, Kroemer G (2010). Molecular mechanisms of necroptosis: an ordered cellular explosion. Nat Rev Mol Cell Biol.

[134] Bergsbaken T, Fink SL, Cookson BT (2009). Pyroptosis: host cell death and inflammation. Nat Rev Microbiol.

